# Premature Deaths Among Children with Epilepsy — South Carolina, 2000–2011

**Published:** 2014-11-07

**Authors:** Anbesaw W. Selassie, Dulaney A. Wilson, Angela M. Malek, Janelle L. Wagner, Gigi Smith, Gabriel Martz, Jonathan Edwards, Braxton Wannamaker, Matthew M. Zack, Rosemarie Kobau

**Affiliations:** 1 Department of Public Health Sciences, Medical University of South Carolina; 2 College of Nursing, Medical University of South Carolina; 3 Department of Neurology, College of Medicine, Medical University of South Carolina; 4 Division of Population Health, National Center for Chronic Disease Prevention and Health Promotion, CDC

Epilepsy is a common childhood neurologic disorder. In 2007, epilepsy affected an estimated 450,000 children aged 0–17 years in the United States (1). Approximately 53% of children with epilepsy and special health care needs have co-occurring conditions (2), and only about one third have access to comprehensive care (3). The few studies of mortality risk among children with epilepsy as compared with the general population generally find a higher risk for death among children with epilepsy with co-occurring conditions but a similar risk for death among children with epilepsy with no co-occurring conditions (4). However, samples from these mortality studies are often small, limiting comparisons, and are not representative (4). This highlights the need for expanded mortality surveillance among children with epilepsy to better understand their excess mortality. This report describes mortality among children with epilepsy in South Carolina during 2000–2011 by demographic characteristics and underlying causes of death. The overall mortality rate among children with epilepsy was 8.8 deaths per 1,000 person-years, and the annual risk for death was 0.84%. Developmental conditions, cardiovascular disorders, and injuries were the most common causes of death among children with epilepsy. Team-based care coordination across medical and nonmedical systems can improve outcomes and reduce health care costs for children with special health care needs (5), but they require more study among children with epilepsy (6,7). Ensuring appropriate and timely health care and social services for children with epilepsy, especially those with complications, might reduce the risk for premature death. Health care providers, social service providers, advocacy groups and others can work together to assess whether coordinated care can improve outcomes for children with epilepsy.

To assess the burden of premature mortality among children with epilepsy, statewide data in South Carolina were analyzed. Four data sources were used: hospital discharges, emergency department visits, hospital-based outpatient clinics, and multiple-cause-of-death data during 2000–2011. Providers in South Carolina are required to submit selected health care encounter data to the state Office of Research and Statistics for planning, intervention, and evaluation of health programs and to support studies related to health and socioeconomic issues in the state.* This office created a unique identifier for children with epilepsy to make it possible to link these data sources while preserving confidentiality (8). The unique identifier was used to identify children with epilepsy across encounters over the course of the study. The probability that two persons had the same unique identifier or a single person had more than one unique identifier is extremely low (8). Duplicate counts for the same encounter were excluded, whereas repeat encounters on different dates were preserved.

Epilepsy was ascertained using diagnosis codes based on the *International Classification of Diseases, Ninth Revision, Clinical Modification* (ICD-9-CM) for epilepsy (345.0, 345.1, 345.3–345.9) and for seizures not otherwise specified (ICD-9-CM 780.39). The positive predictive value of this group of diagnostic codes for an epilepsy diagnosis in children is 96.5% (95% confidence interval [CI] = 88.1%–99.0%) (9). For each case, these diagnosis codes had to be present two or more times within a year, or current procedure terminology codes had to strongly suggest an epilepsy diagnosis (for example, the occurrence of epilepsy treatments such as a ketogenic diet or epilepsy surgery).

Causes of death for children with epilepsy were identified using underlying causes of death grouped by ICD-10 codes. Categorical variables were described using frequencies and proportions and continuous variables using medians and their CIs to minimize the effect of outliers. Children with epilepsy who died were compared with children with epilepsy alive at the end of follow-up by comparing their proportions, medians, or mortality rates, assuming independent samples. In this study, the median durations of follow-up and their CIs distinguish those who died and those who remained alive, characterize the current relative percentages of different causes of death, and allow comparisons with future studies of mortality and the effects of interventions among these children and among other children with epilepsy. All reported differences are statistically significant at a two-sided significance level of p<0.05.

The sum of years from the date of diagnosis or the year 2000 (whichever was later) to the date of death or the end of follow-up and data collection (December 31, 2011) provided person-year denominators to estimate mortality rates per 1,000 person-years by age group, sex, and race/ethnicity. Both the overall risk for death from the follow-up duration and the mortality rate were estimated for children with and without epilepsy (10). The annual mortality rates, age-adjusted to the 2000 U.S. population from 2000 through 2011, were plotted and tested for a linear trend using the Cochran-Armitage test. CIs for these rates were calculated assuming the observed deaths were distributed according to the Poisson distribution.

Two underlying causes of death, developmental conditions (i.e., congenital malformations, chromosomal abnormalities, intellectual disability, cerebral palsy) and cardiovascular disorders excluding congenital malformations, accounted for 30% of the deaths among children with epilepsy (Table 1). Cardiovascular disorders that excluded congenital cardiac malformations (mostly unspecified rheumatic heart disease) were more likely to cause death among older children, whereas other infective heart disease (e.g., infective pericarditis) was more likely to cause death among younger children. Unintentional and undetermined injuries accounted for 11% of deaths among children with epilepsy, 24% of which were side effects of exposure to therapeutic drugs and 16% of which were traffic injuries. Approximately 8% of deaths were caused by epilepsy-specific causes (e.g., status epilepticus).

During 2000–2011, a total of 13,099 children with epilepsy aged 0–18 years were identified (Table 2). These children were followed for a median follow-up period of 38 months (CI = 37–38 months) after diagnosis; median follow-up for the 447 (3.4%) who died was 17 months (CI = 15–21 months), and median follow-up for those who lived was 38 months (CI = 38–39 months). The overall mortality rate was 8.8 deaths per 1,000 person-years. The annual risk for death among children with epilepsy was 0.84% compared with 0.22% among children in the same age groups without epilepsy. The median age at diagnosis for the total cohort was 8 years.

Children with epilepsy who died did not differ from those who survived with respect to age at diagnosis, sex, race/ethnicity, and place of residence (Table 2). Although non-Hispanic blacks represented 29.0% of the state population, they accounted for 38.0% of the children with epilepsy and 41.4% of those who died. Children with epilepsy who died, however, were more likely to have Medicare as their primary health insurance payer (8.7% compared with 3.1%) and less likely to be uninsured (5.8% compared with 12.2%).

Deaths per 1,000 person-years indicate some differences by race/ethnicity, but not by sex, across age groups (Table 3). Among non-Hispanic whites, the mortality rate among children aged 0–5 years (9.8) significantly exceeded that among children aged 6–12 years (5.7). Among non-Hispanic blacks, the mortality rate among adolescents aged 13–18 years (12.4) significantly exceeded that among children aged 0–5 years (7.4).

Annual age-adjusted mortality rates increased from 2000 through 2008, ranging from 2.1 to 5.6 per 100,000 (p = 0.015). But annual rates then decreased to 3.1 per 100,000 in 2011 (Figure).

## Discussion

Epilepsy is one of the most common neurologic disorders in children and can vary widely in its severity and impact (1,2,6). Children with epilepsy are more likely to live in lower-income households and have higher levels of unmet medical needs, mainly because of lack of access to specialized care (1,3). More than one third of deaths among children with epilepsy in this study resulted from developmental conditions and brain disorders, including epilepsy-related causes. About one in nine deaths were associated with injuries. The higher risk for death among children with epilepsy and the higher burden of nonepilepsy-related causes of death supplement findings demonstrating higher risk for death among children with epilepsy with co-occurring conditions (4). Although some causes of death among children with epilepsy were associated with genetic disorders that are not yet preventable, other underlying disorders contributing to cause of death can be better managed with coordinated care (5), potentially reducing excess mortality risk.

Strengths of this study include the use of administrative data facilitating use of standardized diagnostic codes to identify and track large numbers of cases over time. The large sample size permitted subgroup analyses of mortality risk and found few differences by selected epilepsy-related factors or sociodemographic factors. Because children with complex health needs and associated impairments are more likely to be eligible for Medicare coverage, this might explain the higher rate of death among children with epilepsy with Medicare. Although epilepsy-related deaths were not as common as other causes, this study could not assess the level of seizure control, the quality of epilepsy treatment, and treatment complications among children with epilepsy with co-occurring conditions, all of which require further study to identify prevention opportunities.

Although the increased annual death rates through 2008 resulted from an increased case detection rate since the initiation of the study, the 44% decline from 2008 to 2011 is notable. The 2-year delay in documenting all deaths in these datasets could explain the reduced death rate in 2011 but not the reduced rate in 2010. Ascertaining further deaths occurring in 2011 but unreported until later would validate this explanation.

What is already known on this topic?

Children with epilepsy might have an increased risk for death compared with children without epilepsy.

What is added by this report?

Analysis of administrative data from several sources showed that among children with epilepsy in South Carolina during 2000–2011, the overall mortality rate was 8.8 deaths per 1,000 person-years and the annual risk for death was 0.84% compared with 0.22% among children of the same ages without epilepsy. Developmental conditions, cardiovascular disorders, and injuries were the most common causes of death among children with epilepsy.

What are the implications for public health practice?

Ensuring appropriate and timely health care and social services for children with epilepsy, especially those with complications, might reduce the risk for premature death. Health care providers, social service providers, advocacy groups and others interested in improving outcomes for children with epilepsy can work together to assess whether coordinated care for these children can prevent complications associated with epilepsy and reduce their risk for premature death.

The findings in this study are subject to at least four limitations. First, because administrative data designed for billing purposes were the main data sources, certain hospitals might have underreported cases of epilepsy, lowering overall mortality rates from epilepsy in this study. Second, Hispanics accounted for 5.7% of the South Carolina population in 2010, and the 2.2% of deaths among children with epilepsy of Hispanic ethnicity likely underestimates the actual percentage because of coding errors or lack of information on Hispanic patients at free clinics where uninsured migrant farm workers get their medical care. Third, because the study did not consider duration of epilepsy before the start of follow-up in 2000, person-year calculations did not account for earlier years. Finally, causes of death might have been misclassified.

Ensuring appropriate and timely health care and social services for children with epilepsy, especially those with complications, might reduce the risk for premature death. Health care providers, social service providers, advocacy groups, and others interested in improving outcomes for children with epilepsy can work together to assess whether coordinated care for these children can prevent complications associated with epilepsy and reduce their risk for premature death (5–7).

## Figures and Tables

**FIGURE:**
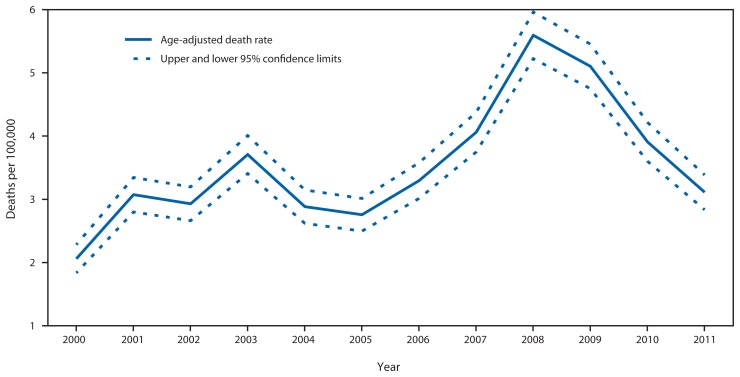
Age-adjusted death rate per 100,000 children aged 0–18 years with epilepsy, by year — South Carolina, 2000–2011

**TABLE 1:** Distribution of underlying causes of death among 447 children aged 0–18 years with epilepsy — South Carolina, 2000–2011

Grouped underlying causes of death (ICD-10 codes)*	%	(95% CI)
Congenital, chromosomal, ID, cerebral palsy (Q00–Q99,F70–F79,R50–R69,G80–G83)	17.5	(13.9–21.0)
Cardiovascular disorders without congenital malformation (I00–I69)	12.8	(9.6–15.9)
Unintentional and undetermined injuries (V01–X59, Y10–Y34, S00–T88)	11.0	(8.0–13.9)
All other causes	10.7	(7.8–13.7)
Disorders of the brain and nervous system (G90–G99)	9.0	(6.3–11.6)
Sepsis or pneumonia (A30–A49, J10–J18)	8.7	(6.1–11.4)
Epilepsy; status epilepticus; seizure, unspecified (G40, G41, R56.8)	8.3	(5.7–10.9)
Malignant neoplasms (C00–C97, D37–D48)	7.2	(4.7–9.6)
Endocrine or metabolic (E00–E90)	4.9	(2.9–7.0)
Suicide or homicide (X60–X84, X85–Y09, Y85–Y89)	4.3	(2.3–6.2)
Liver and digestive disorders (K00–K93)	2.9	(1.3–4.5)
Genitourinary disorders (N00–N99)	2.9	(1.3–4.5)

**Abbreviations:** ID = intellectual disability; ICD-10 = *International Classification of Diseases, 10th Revision*; CI = confidence interval.

* For comparison, the leading causes of death among children aged 0–18 years who did not have epilepsy in South Carolina during 2000–2011, by cause of death (ICD-10 codes) and total number of deaths (n = 9,756, excluding children with epilepsy) included congenital, chromosomal, ID, and cerebral palsy, 12.2%; cardiovascular disorders without congenital malformation, 3.0%; unintentional and undetermined injuries, 25.0%; all other causes, 41.7%; disorders of the brain and nervous system, 2.2%; sepsis or pneumonia, 2.0%; epilepsy, status epilepticus, seizure, unspecified, 0%; malignant neoplasms, 3.5%; endocrine or metabolic, 1.2%; suicide or homicide, 6.9%; liver and digestive disorders, 1.8%; and genitourinary disorders, 0.5%.

**TABLE 2:** Characteristics of children with epilepsy, by mortality status — South Carolina, 2000–2011

			Mortality status			
		
	Total (N = 13,099)		Deceased (n = 447)		Alive (n = 12,652)	
			
Characteristics	%	(95% CI)	%	(95% CI)	%	(95% CI)
**Age group at diagnosis (yrs)**						
0–5	41.7	(40.9–42.5)	39.4	(34.8–44.1)	41.8	(40.9–42.7)
6–12	24.8	(24.1–25.6)	22.6	(23.4–31.9)	24.9	(24.1–25.7)
13–18	33.5	(32.7–34.3)	38.0	(33.5–42.7)	33.3	(32.9–34.5)
Median age (95% CI)	8	(8–9)	10	(8–11)	8	(8–9)
**Sex**						
Male	51.2	(50.3–52.1)	53.7	(48.9–58.4)	51.2	(50.3–52.1)
Female	48.8	(47.9–49.7)	46.3	(41.6–51.1)	48.8	(47.9–49.7)
**Race/Ethnicity**						
White, non-Hispanic	58.4	(57.6–59.2)	56.4	(51.6–61.0)	58.5	(57.6–59.4)
Black, non-Hispanic	38.0	(37.2–38.8)	41.4	(36.8–46.1)	37.9	(37.1–38.8)
Hispanic	3.6	(3.3–3.9)	2.2	(1.1–4.1)	3.6	(3.3–3.9)
**Primary insurance payer**						
Commercial	34.4	(33.6–35.2)	30.0	(25.8–34.5)	34.6	(33.8–35.4)
Medicaid	50.3	(49.4–51.2)	55.5	(50.7–60.2)	50.1	(49.2–51.0)
Medicare	3.3	(3.0–3.6)	8.7	(6.3–11.7)	3.1	(2.8–3.4)
Uninsured	12.0	(11.4–12.6)	5.8	(3.8–8.4)	12.2	(11.6–12.8)
**Place of residence**						
Rural	36.3	(35.5–37.1)	38.5	(34.0–43.0)	36.2	(35.4–37.0)
Urban	63.7	(62.9–64.5)	61.5	(57.0–66.0)	63.8	(63.0–64.6)
**Length of follow-up (mos)**						
* Median (95% CI)*	*38*	*(37–38)*	*17*	*(15–21)*	*38*	*(38–39)*
**Total person-years** *	**50,787**		**984**		**49,803**	

**Abbreviation:** CI = confidence interval.

* The sum of the number of years from the date of diagnosis or the year 2000 (whichever was later) to the date of death or to the end of follow-up, as of December 31, 2011.

**TABLE 3:** Deaths per 1,000 person-years in children with epilepsy, by sex, race/ethnicity, and age group — South Carolina, 2000–2011

	Age group (yrs)							
		
	0–5		6–12		13–18		0–18	
				
Characteristic	Rate	(95% CI)	Rate	(95% CI)	Rate	(95% CI)	Rate	(95% CI)
**Overall**	**8.7**	**(7.4–10.0)**	**7.5**	**(6.1–9.1)**	**10.0**	**(8.5–11.6)**	**8.8**	**(8.0–9.7)**
**Sex**								
Male	8.2	(6.6–10.0)	8.2	(6.2–10.5)	11.3	(9.1–14.0)	9.1	(8.0–10.3)
Female	9.2	(7.3–11.5)	6.8	(4.9–9.1)	8.9	(7.1–11.0)	8.5	(7.4–9.7)
**Race/Ethnicity**								
White, non-Hispanic	9.8	(8.0–11.8)	5.7	(4.1–7.6)	9.0	(7.3–11.0)	8.4	(7.4–9.5)
Black, non-Hispanic	7.4	(5.7–9.5)	10.5	(7.9–13.8)	12.4	(9.7–15.7)	9.7	(8.3–11.2)
Hispanic	6.6	(2.4–14.4)	8.4	(1.7–24.5)	2.5	(0.1–14.1)	6.0	(2. 9–11.1)

**Abbreviation:** CI = Poisson confidence interval.

## Footnotes

* Additional information available at http://rfa.sc.gov/healthcare/dataoversight.
